# The Rise of Non-Tuberculosis Mycobacterial Lung Disease

**DOI:** 10.3389/fimmu.2020.00303

**Published:** 2020-03-03

**Authors:** Champa N. Ratnatunga, Viviana P. Lutzky, Andreas Kupz, Denise L. Doolan, David W. Reid, Matthew Field, Scott C. Bell, Rachel M. Thomson, John J. Miles

**Affiliations:** ^1^The Australian Institute of Tropical Health and Medicine, James Cook University, Cairns, QLD, Australia; ^2^Centre for Molecular Therapeutics, James Cook University, Cairns, QLD, Australia; ^3^Faculty of Medicine, University of Queensland, Brisbane, QLD, Australia; ^4^Immunology Department, QIMR Berghofer Medical Research Institute, Brisbane, QLD, Australia; ^5^Centre for Tropical Bioinformatics and Molecular Biology, James Cook University, Cairns, QLD, Australia; ^6^Immunology Department, Gallipoli Medical Research Institute, Brisbane, QLD, Australia

**Keywords:** Non-tuberculous mycobacteria, pulmonary infection, mycobacteria, immunology, mycobacteria pathology

## Abstract

The incidence and number of deaths from non-tuberculous mycobacterial (NTM) disease have been steadily increasing globally. These lesser known “cousins” of *Mycobacterium tuberculosis* (TB) were once thought to be harmless environmental saprophytics and only dangerous to individuals with defective lung structure or the immunosuppressed. However, NTM are now commonly infecting seemingly immune competent children and adults at increasing rates through pulmonary infection. This is of concern as the pathology of NTM is difficult to treat. Indeed, NTM have become extremely antibiotic resistant, and now have been found to be internationally dispersed through person-to-person contact. The reasons behind this NTM increase are only beginning to be elucidated. Solutions to the problem are needed given NTM disease is more common in the tropics. Importantly, 40% of the world's population live in the tropics and due to climate change, the Tropics are expanding which will increase NTM infection regions. This review catalogs the global and economic disease burden, at risk populations, treatment options, host-bacterial interaction, immune dynamics, recent developments and research priorities for NTM disease.

## Introduction

Non-tuberculous mycobacteria (NTM) are ubiquitous, free living, environmental saprophytic organisms known to occupy water systems, soil, and vegetation. Belonging to the genus *Mycobacterium* (which include *Mycobacterium tuberculosis* (TB) and *Mycobacterium leprae*), there are over 170 identified NTM species with new species discoveries increasing yearly (1). NTM are microaerobic organisms which grow in 6–12% oxygen and have lipid-rich cell walls and metabolic characteristics that result in a slow doubling time of 20–24 h (1). These organisms can withstand a wide range of environmental temperatures, do not readily grow in standard bacterial culture media and are antibiotic and disinfectant resistant. Given these characteristics, NTM are found worldwide and cause infections that are easily missed, difficult to diagnose, and difficult to treat.

First described in the late nineteenth century (soon after Robert Koch's seminal description of *M. tuberculosis* as the causative agent of tuberculosis in 1882), decades passed before human NTM infection was identified (2). Since then over 90 species have been identified from human samples with several more remaining either unclassified or unidentified (3). NTM can be split into “slow” or “rapid” growers. An easy way to narrow down the species in the diagnostic setting. Species classification based on 16S rRNA sequencing has revealed a great deal of complexity within the genus. Human infection is mostly caused by the slow growing *Mycobacterium avium* complex (MAC) which now includes MAC subspecies *silvaticum*, subspecies *hominissuis*, and subspecies *paratuberculosis, Mycobacterium intracellulare, Mycobacterium arosiense, Mycobacterium chimera, Mycobacterium columbiense, Mycobacterium marseillense, Mycobacterium timonense, Mycobacterium bouchedurhonense*, and *Mycobacterium ituriense* (1). Other common NTM isolated from human samples include *Mycobacterium xenopi, Mycobacterium fortuitum* complex, *Mycobacterium kansasii*, and the rapidly growing *Mycobacterium abscessus* group (MABS) which were recently grouped as a separate clade named *Mycobacteriodes abscessus* based on phylogenetic characteristics (4). The MABS group includes subspecies *abscessus sensu stricto*, subspecies *massiliense* and subspecies *bolletii* (3, 5). Collectively, these species comprise 80% of global clinical specimens (3).

The natural habitats for NTM range from natural brackish and marshy waters to municipal water distribution systems and household plumbing including shower heads (6). NTM are also found in potting soil and other peat rich soils. This overlap of bacterial habitat with human habitation provides an ideal opportunity for human infection. The lipid-rich hydrophobic cell walls of these organisms are ideal for biofilm formation which allows long-term persistence of bacterial colonies that are effectively resistant to disinfectants and generate aerosols, particularly from shower heads (7, 8). Organism density in shower aerosols is significantly higher than in the main water stream and is thought to be the most likely source for pulmonary infection (1, 9). Household based studies have shown a genotype match between environmental and clinical isolates (8) while a recent large scale study with multicentre sampling performed in both Europe and the US showed a high degree of overlap between geographical areas where NTM lung disease is common and a high density of potentially pathogenic organisms in shower and water source samples (10). Disturbingly, NTM have also been identified in hospital ice machines, water-cooling systems and haemodialysis unit water supplies. Exposure to these organisms is therefore likely to occur at home to healthcare centers (1, 2). Alarmingly, recent data has confirmed person-to-person transmission of highly virulent, clonal MABS across the globe (11).

## The Pathology of Pulmonary NTM Infection

NTM disease presents a wide variety of clinical syndromes, from lymphadenopathy (commonly cervical lymph nodes) to aseptic meningitis. Infection of the lung is the most common clinical manifestation. Termed pulmonary NTM disease (PNTM), this manifestation has an evolving and complex pathology. Many questions remain including the mode of transmission, the period of incubation and the true disease burden. Three forms of PNTM are described based on distinct pathology. The three forms comprise fibro-cavitary disease, nodular bronchiectasis disease, and hypersensitivity pneumonitis. Given the generally low virulence of these organisms together with their slow growth rate, onset of disease symptoms is often insidious. Incubation periods can vary from months to years making diagnosis difficult and tracing the source of infection virtually impossible. A rise in the number of globally documented NTM infections has led to NTM being recognized as emerging threat causing significant morbidity and mortality in both immune competent and immune compromised populations (12). MAC and MABS are the most common organism groups causing PNTM worldwide (13, 14).

### Risk Groups for NTM Disease

**Figure 1:**
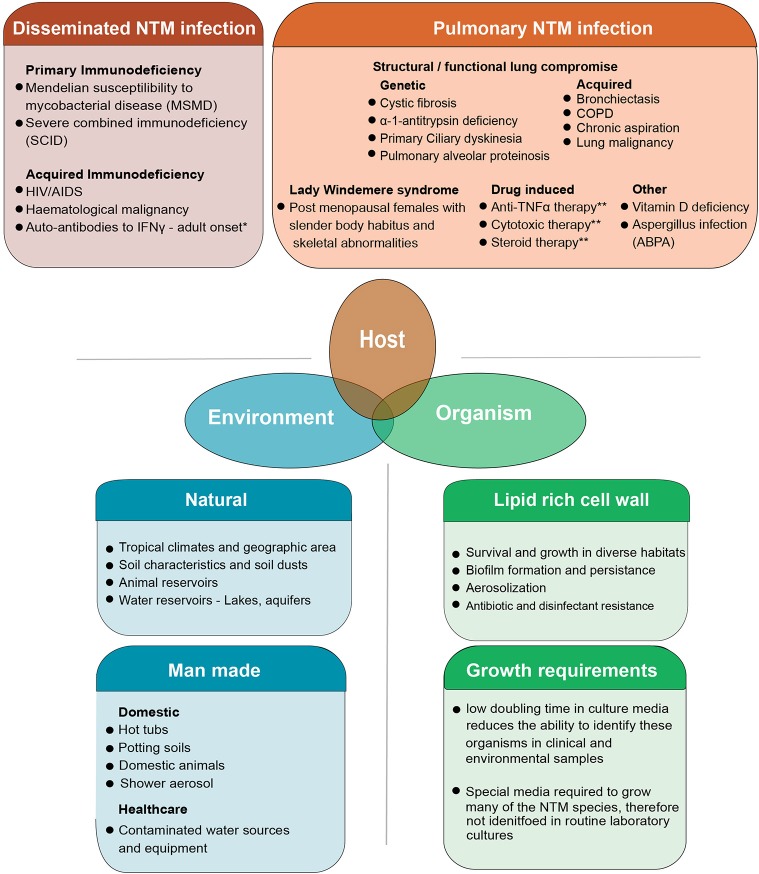
The combined host, environmental and organism risk factors that contribute to developing NTM disease. NTM disease can manifest as pulmonary infection or the more severe disseminated form of the disease which is seen in patients with some severe systemic immune compromise. Pulmonary infection is seen in patients who have structural or functional lung defects that lead to innate immune compromise as well as other groups of patients in whom the precise nature of immune compromise is not clearly defined. Some degree of overlap exists in these risk groups with some patients with systemic immune compromise presenting with pulmonary disease well (15). Environmental risk factors include the natural and man-made habitats where these organisms survive and thrive. Increasing overlap between human habitation and NTM habitats is postulated as a reason for the increasing trend in infection. Organism biology also contributes to infection. NTM are a diverse group of organisms, tolerant to a wide range of physical conditions. Their lipid rich cell wall facilitates biofilm formation and aerosolization of bacteria while simultaneously mediated inherent resistance to many antibiotics and disinfectants. This makes both removing organisms from the man made habitats like water pipes as well as treating patients with active infection, difficult. The specific requirements needed to isolate these organisms in laboratory cultures has meant that NTM are often missed in routine sampling. Though not directly a risk factor for developing infection, this is one of the reasons infections are often missed at early stages. ^1^Autoantibodies to IFNγ are commonly seen in in adults and have been extensively described in East Asian populations. A genetic component to auto antibody formation is likely with specific HLA types being associated with the disease. Both DNTM and PNTM disease manifestations are observed. ^2^Pulmonary alveolar proteinosis has a genetic-based form and acquired form. The genetic-based form is due to gene mutation in GM-SCF subunits and the acquired form is due to auto-antibodies against GM-CSF. This results in impaired surfactant disposal which accumulates in the lung and macrophages leading to dysfunction. ^3^Patients on anti-TNF therapy and cytotoxic therapy are predisposed to both PNTM and DNTM though lung disease is more common. COPD, Chronic Obstructive Pulmonary disease; ABPA, Allergic Broncho Pulmonary Aspergillosis.

NTMs are considered opportunistic pathogens to humans. Exposure to these organisms in day-to-day life is common through shower aerosols but infection and clinical disease occur in only some individuals (8). Over the last decades it has become apparent that several groups of individuals are prone to PNTM disease (Figure 1). These include patients with both genetic or acquired structural lung diseases such as cystic fibrosis (CF), chronic obstructive pulmonary disease (COPD), non-CF bronchiectasis, alpha-1 antitrypsin deficiency, previous pulmonary tuberculosis, and lung cancer (16–18). Patients with immune suppression due to primary immune deficiency syndromes (PIDs) such as Mendelian Susceptibility to Mycobacterial Disease (MSMD) associated with IL12-p40, IL12, IFNγ receptor abnormalities and gene deformities (IFNγR1, IFNγR2, IL12RB1, IL12B, STAT1, IKBKG, CYBB, ISG15, IRF8, GATA2) are at high risk of NTM infection (19–21). In addition, patients with acquired immunodeficiency syndromes including AIDS and hematological malignancies, hairy cell leukemia in particular, are also identified as susceptible to NTM infection (22). The latter groups of patients however, usually develop disseminated NTM infection (DNTM) rather than isolated pulmonary NTM infection (PNTM) which is seen in patients with structural lung disease and are considered a separate risk group (Figure 1). Other acquired states of immune deficiency, such as haematopoietic stem cell transplantation and solid organ transplantation are also predisposed to NTM infection. However, these patients could present with PNTM, DNTM, or other extra pulmonary sites of NTM infection (23). Other specific PIDs like Severe Combined Immune Deficiency (SCID) are commonly associated with BCGiosis, while Common Variable Immune Deficiency (CVID) predisposes patients to bronchiectasis which, in-turn, can lead to PTNM infection (21).

The increase in research into the epidemiology, diagnostics, and treatment of this once obscure disease stems from the increasing numbers of cases being identified from populations with previously unknown and currently unidentified risk factors (12). Advances in therapeutics in all fields of medicine have seen unexpected NTM disease susceptibilities emerge which pose a challenge in terms of patient care but also provide insight into disease pathology. For example, the susceptibility of patients with rheumatoid arthritis on anti-TNF therapy (infliximab, adalimumab, golimumab, and certolizumab) to NTM infections is a prime example of unexpected NTM susceptibility (24–26). These patients commonly present with PNTM disease though extra pulmonary manifestations are also common. DNTM infections are rare though they have been described (27).

A fourth disease cohort include elderly white post-menopausal females who present classically with NTM infection of the middle or lingular lobe of the lung. Described as “Lady Windermere syndrome” these patients often have a distinct physical phenotype of slender build, pectus excavatum or scoliosis and mitral valve prolapse, though notably they have no known immune dysfunction (16, 19, 28). Recently identified genetic defects that could contribute to susceptibility in these “Lady Windemere” patients include cystic fibrosis transmembrane conductance regulator gene (CFTR) related mutations, ciliary function, and other connective tissue related genetic defects as well as the DNA damage response protein TTK defects (22, 29–31). Finally, gastro-esophageal reflux disease (GORD), vitamin D deficiency, rheumatoid arthritis (26, 32, 33) and low body mass index (BMI) are art risk of NTM lung disease (34). The acquire and genetic risk factors for NTM infection, both PNTM and DNTM are discussed in a recent reviews by Honda et al. (35) and Henkle et al. (23) showing the many forms and known susceptibilities the disease takes.

### The Global Disease Burden of NTM

Studies from North America, Europe, and Asia have all shown increasing NTM disease incidence over the last two decades. Estimated NTM disease prevalence rose from 2.4 cases/100,000 in the early 1980s to 15.2 cases/100,000 in 2013 in the US (36). The prevalence in the elderly population (>65 years) more than doubled from 20 cases/100,000 to 47 cases /100,000 population between 1997 and 2007 (37). Multiple studies in five US states showed NTM positive culture rates increased from 8.2 cases/100,000 in 1994 to 16 cases/100,000 in 2014 (38). Similar figures are recorded in a Canadian study published in 2017 with disease prevalence increasing from 4.65 cases/100,000 in 1998 to 9.08 cases/100,000 in 2010. Laboratory isolation rate increased from 11.4 isolates/100,000 in 1998 to 22.22 isolates/100,000 in 2010 (39). The prevalence of NTM disease in non-cystic fibrosis (NCF) bronchiectasis in the US is estimated as 37% with the most common isolate being MAC (37). Laboratory isolation of NTM are now more common than *M. tuberculosis* in the US and Canada with an increase of 8.4% annually being documented between 1997 and 2003 (17). A study from the UK showed similar increases with the NTM infection rates more than tripling from 0.9 cases/100,000 in 1995 to 2.9 cases/100,000 in 2006 (40). Similar rates have been documented in Denmark (41) and Germany (42).

Studies in South Korea showed a 62% increase in NTM lung disease from 2002 to 2008 with a marked increase in MABS infection (43). This is in contrast to European studies that show a predominance of MAC infection (44, 45) Numbers from Japan have shown a marked increase in both NTM infection and mortality from 1994 to 2010 (46) while a population-based Chinese study showed an increase in NTM isolation rate from 3 to 8.5% from 2008 to 2012 (47). As NTM disease is not a notifiable disease in most countries, accurate epidemiological data is limited, particularly in countries with low development indices. Nonetheless, an increasing number of NTM cases have been recorded in Brazil, Taiwan and the Middle East (48–52).

Globally, the most common NTM pathogens are the MAC organisms though prevalence varies greatly with geographic region, gender, and age (49). MABS are a significant problem particularly because of very high levels of antibiotic resistance and the disease a growing problem in East Asian countries including Japan, Korea, and Taiwan (53). NTM are also a particularly difficult problem in patients with cystic fibrosis, which is the most common genetics disease in Caucasians, whom are highly prone to MABS infection (40).

Cultures from CF patients have an ~10,000-fold higher NTM prevalence compared with the general population (21). NTM isolation rates in CF vary from 3 to 17% with an increase in median prevalence from 9 to 13% seen in pre- and post-millennial studies (54). Increased prevalence of NTM positive cultures is seen with increasing age (55). Prevalence rates in the Australian adult CF population was 4.1% in a 2001–2014 retrospective study carried out in Queensland (56). Though not as common as other bacterial pathogens, NTM infection was recognized as an important clinical entity in these patients as it was associated with significant deterioration in lung function (57). A geographical variance is seen in NTM species prevalent in the CF population, with MABS and MAC remaining the most common PNTM infections in these regions (54). Genetic mutations in CF patients are associated with PNTM (58).

**Figure 2:**
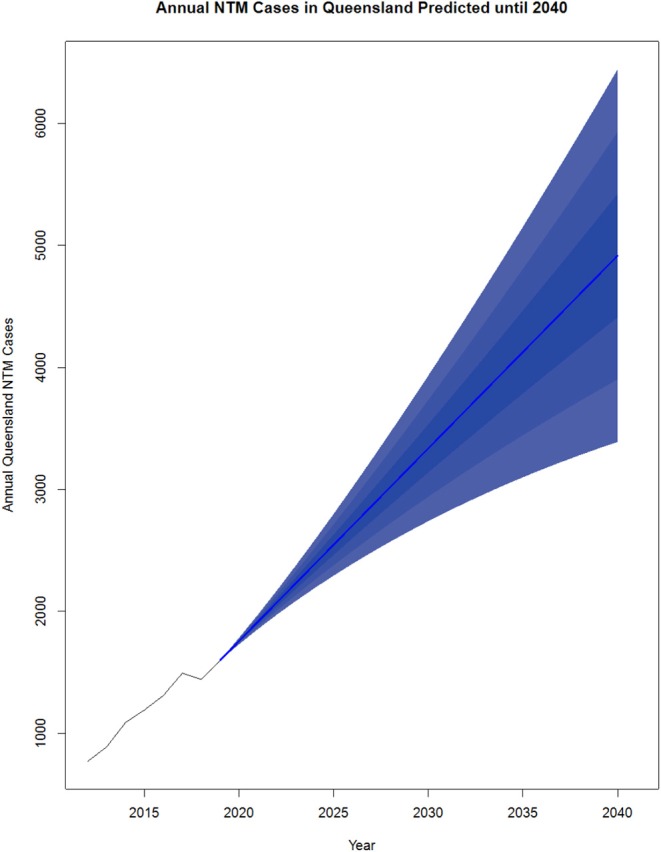
Projected NTM cases in Queensland, Australia from 2020 to 2040. NTM cases from 2012 to 2019 were reported by the Epidemiology and Research Unit, QLD Department of Health and analyzed using R v3.5.2. The existing data was converted to a time series object using data from 2012 to 2019. The R package forecast (65) was used to generate the predictions from 2020 to 2040. The order for the model was estimated using the auto. arima() function which takes in a time series and returns the best AutoRegressive Integrated Moving Average (ARIMA) model according to either AIC, AICc, or BIC value. Each model was input to the forecast function with levels average, 5, 10, and 25 plotted.

NTM pathology has been a notifiable disease in Queensland (QLD), Australia since the commencement of the tuberculosis (TB) control programme in the 1960s and is currently a notifiable disease (59, 60). The increase in disease incidence in QLD over the last several decades has been clearly documented. Clinical cases of MAC disease were reported as 0.63 cases/100,000 in 1985, 1.21 cases/100,000 in 1994 and 2.2 cases/100,000 in 1999 (59). Significant NTM species isolation rates then rose from 9.1 cases/100,000 to 13.6 cases/100,000 from 1999 to 2005. In total, 1,171 isolates were reported in 2016 which is almost double the 672 isolates reported for the same period in 2012 (60). An increase in MABS isolates was also seen during this period. Of note, there was a change in the gender distribution from male predominance in 1999 to female predominance in 2005, particularly in the elderly population (59). Overall, a pattern of increasing non-cavitary disease in elderly females at a rate of 2.2–3.2 cases/100,000 population per year has emerged. Similarly, an increase in NTM disease has also been seen in the Northern Territory (NT), Australia from 1989 to 1997 (61). Regarding infection sources, subsequent investigation showed MAC, and MABS were present in household and municipal water sources and shower aerosols in homes (62–64). Projections show cases could more than triple between 2020 and 2040 [up to 6,446 cases a year (CI 15 just in QLD] (Figure 2).

## The Treatment, Complications, and Economic Burden OF NTM

PNTM treatment requires prolonged (12–18 months) multi-drug therapy (66). Disease remission rates vary depending on infecting species, patient age and comorbidities (37, 67). Recurrence is common with rates of 30–50% being recorded in MAC infection (68). The majority of these recurrences are due to reinfection (69, 70) as opposed to relapse. MABS infection is more likely to result in treatment failure and recurrence. Many patients develop persistent chronic infection despite treatment while others succumb to the disease (5, 67). Side effects of antibiotics are numerous, and regimes are difficult to tolerate. Treatment is at high cost (USD $14,730 for MAC infection and USD $47,240 for MABS infection) (67). Of concern, long term treatment with multiple antibiotics increases antibiotic resistance and there is now evidence of person-to-person transmission of NTM (67). A multicentre study of MAC infection across Canada, France, Germany and the UK conducted in 2018 showed average direct medical costs per person year ranged from $US12,200 in Canada to $US25,500 in France (71). In addition to direct disease related costs, patients were also shown to have six times higher secondary care utilization events for disease-related and disease-unrelated illnesses (18).

Adjuvant therapies have been tested with little success. Preliminary trials of adjunctive IFNγ therapy were abandoned due to lack of response (72–74) although early case studies performed in patients with refractory disease showed promise (75, 76). IFNγ therapy (by intramuscular injection, as opposed to the original trials done with nebulized IFNγ) showed promise in a recent study (77) but no other studies have supported these results (34). Other immune modulatory agents tested include recombinant IL-12 in mice (78, 79) and GM-CSF in HIV infected patients (80, 81). A phase 2 open labeled drug trial is currently underway to test the efficacy of inhaled GM-CSF in persistent NTM infection (NCT03421743).

### The Host-Bacterial Interaction

NTM are not classic species-specific pathogens, rather they are environmental saprophytic organisms that make use of the new living opportunity presented when human habitation and bacterial habitation overlap. Unknowns include: (i) the percentage of a given population who are exposed; (ii) how infection occurs and by what source; (iii) what host and bacterial factors determine clearance; (iv) how NTM establishes itself as a colonizer without causing tissue invasion and; (v) why NTM are symptomatic in only some individuals. All that is currently known is that specific groups of individuals are at risk, some with known immune dysfunction, and others with specific medical characteristics.

### The Immune Response in Pulmonary NTM Infection

The immune responses seen in human NTM infection has shown similarities to TB. However, no consistent phenotype of immune protection or immune susceptibility has been described. Immune compromise caused by genetic mutations (MSMD) and acquired defects due to infections like HIV usually lead to disseminated infection while iatrogenic causes (inhaled or systemic corticosteroids, anti-TNF therapy, chemotherapeutic agents), and defects in lung structural and functional integrity (primary ciliary dyskinesia and other mutations leading to ciliary dysfunction, CFTR mutations, bronchiectasis, COPD, α1 anti-trypsin deficiency, lung malignancy, and ATT) and pulmonary alveolar proteinosis, are known predispositions to pulmonary NTM disease (18, 22, 82). Previous or concomitant TB infection and Aspergillosis independently increase risk of PNTM (83).

These predispositions tell a story of both local/systemic and innate/adaptive immunity being required to combat infection. Innate defense mechanisms such as effective respiratory epithelial ciliary function are likely required to keep colonizing NTM bacterial counts under control. When airway mucociliary clearance is impaired and/or when virulent strains of bacteria can locally invade tissue, cellular defense mechanisms are activated. The immune cascade then follows: (i) macrophage activation and local recruitment of innate cells including neutrophils, iNKTs and NK cells to control early infection and; (ii) migration to of APCs to lymph nodes for antigen presentation and activation of antigen specific T cells. A review by Tomioka (84) describes the cytokines and other factors involved in macrophage activation as well as the key players involved in transforming naïve T cells to either Th1 type or Th2 type during mycobacterial infection. Macrophages and NK cells release IL-12/ IFNγ to guide T cells toward a Th1 type phenotype. Th1 IFNγ and IL-2 release then promote intracellular killing of mycobacteria. The exact triggers for a Th2 type response are not known, but should a Th2 type response predominate, Type2 cytokines (IL-4, IL-10, and IL-13) promote suppressive pathways that increased Treg cell frequency.

Mouse studies have shown that RORγt induced Th17/IL-17 responses during MAC infection promote pulmonary inflammation (85). However, the mechanism/s and correlates of protection of these responses during the various stages of this chronic disease are not understood. Other studies in the mouse models or murine cells models of NTM infection have shown the importance of CCL2, CCL5, and TLR signaling via MAPK, MyD88, and NFκβ for disease protection (86–88). Robust mouse models for *M. avium* infection exist though currently, it is difficult to initiate infection, maintain infection, and measure immune responses in MABS mouse models (89, 90). While comparisons between immune competent and immune deficient mouse models have provided insight into immune dysfunctions associated with DNTM (90), the chronic stages of PNTM infection, which are of current clinical relevance are not yet fully reproducible in mice.

Laboratory and clinical studies of mycobacteria immunity have shed light on some aspects of why opportunistic infections occur. Most studies have either measured cytokine levels directly in serum or cell culture supernatants where cell preparations have been stimulated with antigens or other non-specific mitogens such as lipopolysaccharide (LPS), which activates myeloid cells, or phytohaemaglutinin (PHA), which stimulates cellular immunity. A comparison of MAC infected patients with no evidence of compromised immunity and *M. avium* sensitin skin test positive healthy controls, showed that infected patient peripheral blood mononuclear cells (PBMCs) stimulated with mycobacterial antigens produced higher levels of IL-10 but lower levels of IFNγ, IL-12, and TNF. Other studies have shown similar results for IFNγ and IL-10 but not for the other cytokines (91–94). A study of serum cytokine levels comparing newly diagnosed MAC patients showed a significant reduction in IL-6, IL-8, IL-23, IFNγ, and CD40L (95). Longitudinal assessment of Th1 and Th17 cytokines in these patients after 1 year of antibiotic therapy showed that while low Th1 cytokine levels could accelerate infection, Th17 cytokine levels at diagnosis (IL-17 and IL-23) could act as indicators of treatment outcome (sputum conversion vs. failure). A comparison of immune responses in MAC and MABS infection showed that MABS stimulated PBMC produced higher levels of TNF, IFNγ, IL-1β, and MIP-1α than MAC stimulated PBMC (96). A study that compared IFNγ, IL-12, and IL-10 production in response to mitogen-stimulated PBMCs in patients with MAC, MABS and healthy controls showed a reduction in IL-10 production in patients (97). A more comprehensive study, that used multiplexed bead-based assays to evaluate 22 cytokines in 24 MABS patients, showed reduced levels of IFNγ, IL-12, IL-4, and IL-13 and high levels of IL-17 and IL-23 in patients. A hi-dimensional flow analysis between individuals at risk and not at risk of MABS disease revealed immune exhaustion in T cells (CTLA-4) may be playing a role (98). These finding is similar to studies performed in MAC infection (93, 95). Interestingly, levels of monokine induced by IFNγ (MIG) and IFNγ induced protein (IP-10) could predict treatment outcome (99). A recent small study on cytokine levels in three CF patients with MABS infection compared to three patients with non-CF PNTM infection and healthy controls showed no difference in TNF and IL-1β levels between CF and non-CF patients, however the non-CF patients showed higher TNF and IL-1β production following LPS stimulation (100). A hi-dimensional flow analysis between CF individual at risk and not at risk of MABS disease revealed a several immune biomarkers with a combined Akaike information criterion (AIC) of −30 and an area under the curve (AUC) of 1 (101). Additionally, the at risk CF patients showed a clear deficiency in TNFα release from both CD4^+^ and CD8^+^ subsets.

Preliminary evidence showed that T cell defects may play a role in MAC infection (102). T cells from healthy control subjects exhibited superior MAC growth inhibition in monocytes compared with patients. A recent study by Shu et al. (103) showed higher PD1 expression in T cell in patients with MAC lung disease compared to controls. This study also showed reduced IFNγ and TNF production in MAC patients which was partially corrected after 2 months of antibiotic treatment and could also be further increased by blocking PD-1. However, this report did not study T cell function. A study using monocyte derived macrophages (MDM) showed no difference in MDM cytokine responses between patients and controls (104) while a more recent study showed that Keap 1 (an oxidative stress sensor) negatively regulated inflammatory signaling from primary macrophages in MAC infection (105). Other studies of TLR and dectin-based signaling in MAC and MABS infections showed TLR signaling to be crucial (96, 104–106). In addition, MAPK signaling, ERK1/2 and p38 have been shown to be down regulated in patients with MABS infection with subsequent reduction in TNF, IL6, and IL10 (107). Similar to studies in TB, different strains of NTM have been shown to elicit different immune responses in both human cells and murine models showing the importance of pathogen genetics on the host response (101, 108).

Studies on human cells have varied in the specimen used [PBMC, broncho-alveolar lavage (BAL) fluid and whole blood], the stimulants used (PHA, LPS, neutralized bacteria) and patient groups (age, infecting species, and stage of treatment) as shown in Table 1, making both cross-study comparisons and interpretation challenging. In addition, patient age ranges often vary widely, including multiple risk groups, and other confounders.

**Table 1:** Summary of immune cytokine profiles during *in vitro* studies of patient immune responses in PNTM infection.

**Study population**	**Patient #**	**Organism**	**Sample**	**Stimuli**	**Measurement**	**Result**	**References**
PNTM patients before or during treatment vs. MTB^a^ patients vs. HC^b^	32	MAC and M. *kansasii*	PBMC supernatant	PHA^c^, anti-CD3, PPD^d^, and viable NTM	Cytokines by ELISA	Patients—↓ IFNγ and TNF	(92)
PTNM patients before or during treatment vs. HC that were MAC sensitin+	26	MAC	PBMC and BAL^f^ supernatant	Heat killed MAC and MTB	Cytokines by ELISA and ICS^h^	Patients—↑ IL10 (produced by T cells and monocytes) and ↓ IFNγ, IL12 and TNF	(90)
PNTM patients with persistent NTM infection vs. HC	5	MAC	PBMC supernatant	PHA, PMA^e^ and anti-CD3	Cytokines by ELISA	Patients—↓ IFNγ	(91)
PNTM patients vs. HC	29	MAC and MABS	PBMC supernatant	PHA +/– IL12 and LPS +/–IFNγ	Cytokines by ELISA	Patients—↓ IFNγ, TNF, and IL12p40^i^	(96)
PNTM patients before or during treatment vs. HC (related) or HC (general population)	17	MAC	PBMC supernatant	SEB^g^, PPD, and MAC sensitin	Cytokines by ELISA and ICS	Patients—↑ IL10, IFNγ, IFNγ+ by CD4+ T cells and ↓ IL17	(93)
PNTM patients before treatment vs. HC	42	MAC	Serum		Cytokine array	Patients - ↓ CD40L, IFNγ, IL6, IL8, and IL23	(94)
PNTM patients vs. HC	50	MAC	PBMC and MoDC supernatant	MAC sensitin, heat killed MAC and PHA	Cytokines by ELISA	Patients -↓ IFNγ and TNF	(102)

a *MTB: Mycobacterium tuberculosis*.

b *HC: Healthy Controls*.

c *PHA: Phytohaemagglutinin*.

d *PPD: Purified Protein Derivative*.

e *PMA: Phorbol myristate acetate*.

f *BAL: Bronchoalveolar lavage fluid*.

g *SEB: Staphylococcal enterotoxin B*.

h *ICS: Intracellular cytokine staining using flow cytometry*.

i *Same result for both MAC and MABS*.

Indirect evidence suggests individuals prone to NTM infection have underlying immune dysfunction. Mutations known to cause susceptibility include those affecting IL12β, IL12Rβ1, IFNγR1, IFNγR2, and transcription factor STAT1 and RORC (109). Deficiency in NFκβ essential modulator (NEMO) and other primary immunodeficiency syndromes like GATA-2 deficiency and isolated CD4^+^ T cell deficiency have also been implicated in NTM susceptibility (21, 110). A recent study showed association between TNFA-1031 and IL10-1082 alleles and NTM infection (111). Additionally, HIV infection increases the risk of NTM disease when CD4^+^ T cell counts drops below 50/mm^3^. Broadly immunosuppressed patients with hematological malignancies, organ transplants, and stem cell transplants are at high risk. The timing of this increased risk does not coincide with the neutropenic phase of these diseases highlighting the lack of importance of neutrophil action in NTM immunity (21). Current available information supports the increased risk of NTM in patients being treated with anti-TNF therapy (24). There is also evidence for increased risk in patients on the anti-IL6 agent tocilizumab while other agents including IL12/IL23 inhibitor ustekinumab (associated with TB reactivation), and the JAK pathway inhibitors tofacitinib and ruxolitinib (associated with IFN signaling interference) pose a theoretical risk. However, robust information is not yet available (21, 25).

NTM disease biomarkers (vs. airway colonization which is commonly seen in chronic lung diseases like CF, COPD, and bronchiectasis) are of high clinical value. Likewise, the identification of patients likely to recover and patients likely to develop serious life-threatening infection would be of enormous benefit to clinicians to guide the therapeutic decision-making process. Information from mouse models of MAC infection are available and less so for MABS. Human information is limited to small studies of generally <10 patients (89). Information is still lacking around the immune profiles of CF patients with MAC and MABS disease in comparison to non-CF patients with disease. Longitudinal follow-up information of the changes seen in the immune profile of these patients during treatment is also not available. In-depth analysis of the immune function and dysfunction seen in these groups of patients will provide much needed insight into disease pathophysiology and ultimately therapeutics (immune modulators etc) that could be developed and/or repurposed to enhance immune responses to these life-threatening infections.

## Recent Developments and Research Priorities

Recent findings of increased NTM pathophysiology are cause for global concern. Firstly, the recent emergence of person-to-person transmission of highly antibiotic resistant MABS across continents is highly alarming (11). These findings have led to new infection control practices in the US, UK, and Australia (34, 55, 112). Secondly, evidence suggests there is increasing incidence of childhood NTM disease. A nationwide, population-based study showed a significant increase in childhood NTM infection following a change in national policy on BCG vaccination from “universal” to “selective” (113). This study suggests that while BCG may provide some degree of protection to children from NTM infection, unvaccinated children, and other populations with respiratory deficits like CF could be a susceptible to this disease. Other studies have documented similar trends, particularly in relation to extra-pulmonary NTM infection in children, support this theory (114). Thirdly, it has been postulated that that MAC infection increases tumor-genes inflammatory responses which could lead to an increased risk of breast and lung cancer (115). Studies have associated NTM infection with diseases such as Sjogren's syndrome in Taiwan (116) and Sweets syndrome in Japan (117), though few, these studies highlight the possibility that NTM infection may catalyze non-infective sequalae that add to morbidity. Fourthly, there are alarmingly high death rates in patients following diagnosis with NTM lung infection. A systematic review showed a 5 years mortality showed 27% in Europe, 35% in the US and 33% in Asia (118). Predictors of high mortality included male gender, presence of comorbidities, and fibro cavitary disease. These findings have been validated in other studies that showed that male patients, with fibro cavitary disease, low BMI and malignancy were prognostic indicators of poor clinical outcome (41, 119, 120). In addition, patients with persistent infection (those who remain culture positive despite 12 months of treatment) have higher rates of death attributable to NTM infection compared to those who manage to clear NTM in the sputum (34). Significantly higher numbers of hospitalizations due to illness, leading to increasing health care costs compound this issue (42).

Research priorities recommended in the US and UK include rapid diagnostic tools fast identification of infecting species (34) and simple and cheap screening tool to identify patients at risk (83, 98, 112). These are considered high impact research goals that would alert clinicians to at risk patients enabling faster initiation of appropriate treatment and ultimately, superior care.

## Discussion

NTM infection presents a growing global health problem, complicated by ubiquitous exposure to the organisms, incomplete understanding of the immune susceptibility to disease, increasing numbers of immune compromised patients, cumbersome diagnostic tests (with no prognostic tests) and costly, multi drug treatment regimens that often fail to cure. However, we must keep in mind that different disease mechanisms may be operating between different risk groups and preclinical models.

NTM disease is frequently slow and progressive, affecting predominantly already vulnerable patient populations. Epidemiological and descriptive studies of patients are many, but gaps in knowledge remain. Foremost among these is a deconstruction of the immune susceptibilities to NTM lung disease. If we can understand potential patient risk profiles, screening tests could be efficiently deployed to identify infection at risk individuals within hours. Such screening tests as well as prognostic tests that can predict outcome (disease remission vs. persistence, optimal treatment course, life changes etc) during early treatment would be extremely beneficial for clinicians to make therapy decisions as soon as possible, with potential improvement of patient outcomes. In the current age of immunotherapy, where targeted augmentation of immune responses is now possible, research into adjuvant immune therapies that could be used to “boost” a weakened immune system would beneficial and could be redeployed from the cancer field. Such immune modulating interventions would go a long way in reducing the global burden of NTM disease.

The true level of morbidity caused by NTM lung disease is slowly being revealed, in both developed and developing nations and in both immune competent and immune compromised populations. Disease burden is being documented in both childhood and adulthood disease in terms of both direct and indirect morbidity. A cohesive solution to the global challenge of NTM lung infection requires a multipronged approach involving not just epidemiological data, but also clinical and laboratory-based research for new diagnostics, prognostics, and treatments for use in machine learning. These cohesive approaches are urgent as NTM is more common in the warmer climates (60). Forty percent of the world's population live in the tropics^1^ and due to climate change, the tropic are expanding in area (121).

## Author Contributions

CR drafted the manuscript. VL, AK, DD, DR, MF, SB, RT, and JM provided critical revision.

### Conflict of Interest

The authors declare that the research was conducted in the absence of any commercial or financial relationships that could be construed as a potential conflict of interest.

## Footnotes

^1^ Available online at: http://worldpopulationreview.com/countries/tropical-countries/. (2019).
